# The dark ventral patch: A bimodal flexible trait related to male competition in red deer

**DOI:** 10.1371/journal.pone.0241374

**Published:** 2020-11-05

**Authors:** Juan Carranza, Eva de la Peña, Concha Mateos, Javier Pérez-González, Susana Alarcos, Jerónimo Torres-Porras, Juliana Valencia, Cristina Sánchez-Prieto, Leticia Castillo

**Affiliations:** 1 Wildlife Research Unit (UiRCP), University of Córdoba, Córdoba, Spain; 2 Biology & Ethology, University of Extremadura, Cáceres, Spain; 3 Department of Social and Experimental Sciences Teaching, Faculty of Educational Sciences, University of Córdoba, Córdoba, Spain; 4 Experimental Sciences Teaching, Faculty of Educational Sciences, University of Málaga, Málaga, Spain; Universita degli Studi di Sassari, ITALY

## Abstract

Sexual signals play a central role in male-male competition in polygynous species. In red deer (*Cervus elaphus*), male’s ventral area become dark during the rutting season due to urine spraying behaviour and retains many chemical compounds potentially revealing individual features. Here we investigate the variation in size of this trait, exploring its relationship with age and male competitive features such as antlers or body size, as well as populational level of intrasexual competition for mates. We found that the size of the dark ventral patch followed a clearly bimodal distribution, i.e. males mostly expressed the full-size trait or just developed a very small one. For these two groups of males according to trait expression, the relationships of trait size with age and antler size differed. Populational level of intrasexual competition appeared to affect the relationship between antler size and the probability of a fully developed ventral patch. These results indicate that the trait encodes information on body size, antler size, age and populational level of mate competition, thus suggesting a role in signalling male’s competitive features and willingness to allocate reproductive effort within a particular season.

## Introduction

Sexual selection promotes the evolution of traits, such as weapons or signals, involved in different modes of competition for mates [1]. Sexually selected traits are costly, especially those favoured by signal selection [2–4]. These costs may be caused by the nature or amount of materials needed to produce the trait, by their effects in social interactions, or by the reduced efficiency of associated traits subjected to different modes of selection (viability, immunocompetence; see [5]).

Sexual traits may be better afforded by good condition individuals, for which the costs of production and maintenance of the traits may be lower [1–3, 6, 7]. But also, on the side of the benefits, selection may pay individuals to assess the potential benefits related to the production of sex traits before incurring in their costs [8].

The flexible expression of sexual characteristics allows to respond in time to ecological changes or seasonal variations in which an individual can be found [9–11]. The success in competition may also be sensitive to variations in the social environment (e.g., the sex ratio). Hence, selection may pay individuals to adaptively adjust trait investment [12, 13]. Subordinate males may reduce investment in reproduction until their chances for increasing rank and mating opportunities raise [11] and the development of appropriate sexual signals may contribute to fitness through interactions with conspecifics.

Some traits are more flexible than others. Some plumage changes in birds, weapons in mammals or body size in many species are produced according to ontogenetic or seasonal temporal patterns but may have little potential for short-term flexibility [11, 14–16]. Other characters, such as those predominately behavioural, have higher potential for being modulated according to the current cost/benefit balance [17–19]. In many cases, when individuals are producing the trait, they may not be aware on the expected cost-benefit balance at the time of competition. For instance, if trait development takes place before the start of the breeding season and thereafter the conditions (e.g. the number of competitors) may change.

While sexual traits have received much attention in research, flexible sex traits have been much less studied. The study of the relationships between sex traits and male's condition or social environment has been approached by correlational work and, in fewer cases, by experiments either under controlled conditions (*Taeniopygia guttata* [18]; *Phasianus colchicus* [20]; *Astatotilapia burtoni* [21]; *Alectoris rufa* [22]) or in the wild (*Mandrillus sphinx* [17]; *Malurus melanocephalus* [11]).

The Iberian red deer (*Cervus elaphus hispanicus*) is a highly polygynous species [23], in which males strongly compete for females either by defending harems or mating territories [24–26]. The most famous sex trait in red deer is the set of antlers. Antlers are renewed every year, so they are a rather flexible trait compared for instance with horns of bovid species, although they are produced several months before the starting of the rutting season. Apart from cues on their own condition to afford trait production, some information on the structure of the population, likely potential mates and number and quality of competitors, may also affect the growth of antlers [27]. However, males and females move from their normal ranges to mating areas during the rutting season, so short-term assessment during the rutting season may become valuable if the individual is able to modulate its competitive traits. This is not already possible with the antlers that were produced some months before, but other traits may be more modulable in the short term.

Red deer males during the rut show a very conspicuous trait as a dark brown, almost black, ventral large patch from the opening of the penis up to the ventral base of the neck. This dark area is produced by urine [28] and it is impregnated by great amount of lipidic compounds [29]. These chemical profiles change with male age [28] and intrasexual competition situation [30]. Our own preliminary analyses show that males with larger patches are more involved in the rut and achieve higher mating success than males with small patches [31]. Additionally, ventral patch size is indicative of DL-3,4-dihidroxyphenil glycol (DOPEG) levels in urine [28]. DOPEG is a metabolite of norepinephrine that reflects high fighting capacity or high ability to escape from predators, favouring individuals to achieve lifetime fitness. Besides its role as a chemical signal, and because there is a clear process producing the staining of the fur of this patch [28], it is also likely that patch size can contribute as a visual signal to the assessment of rivals in intrasexual competition and possible mates in female mating choice.

The main aim of this study was to investigate the size of the dark ventral patch of Iberian male red deer, and its relationships with individual and population features. We expected that the size of the dark patch, as a morphological feature, were related to age, antler length and body size. However, as a sexual signal, trait expression may go beyond the simple relationship with size. Hence, our hypothesis was that males should express a full sized dark ventral patch when they are willing to invest in intrasexual competition, which may relate to individual features such as size, although not necessarily in a linear manner, and also to social conditions such as the number of rival males with respect to available females in the population.

## Material and methods

### Study areas and data

Ethical issues were revised and approved by UIRCP, University of Cordoba, We studied red deer populations under two types of management regimes in hunting estates in Spain that range in size between 750 and 3000 Ha. Under one of these regimes, estates are fenced by 2 m high stock-proof wire mesh, while in the other, areas are unfenced allowing deer free movement between estates [32, 33]. Fenced hunting estates reduce hunting pressure on young males, allowing them to reach maturity. By contrast, in unfenced hunting estates, because there is not a common practice between neighbouring estates sparing young males, few stags reach mature age, as no estate wants to preserve adult stags because they are at risk of being shot when moving to adjacent estates [33]. Thus, on unfenced estates, hunting pressure is not on deer with best trophies, but on almost every male above two years of age, sparing yearlings as it is illegal to shoot them [33]. Population density is similar in both types of populations, averaging 0.3 individuals per hectarea [33]. However, as a consequence of such contrasting management, the sex-ratio and age structure in red deer populations within unfenced areas are strongly biased towards females and young males, compared to the situation in fenced areas [32, 33; mean age of culled males: 2.61 years in unfenced areas compared to 4.51 years in fenced ones; 27]. Therefore, in fenced estates, males experienced a high level of intra-sexual competition compared to that in unfenced estates, where virtually all males can mate even if they are sub-adult [27; 32, 33]. From now on we will refer to fenced and unfenced estates as populations with high (HC) and low (LC) levels of competition for mating opportunities. We considered populations either fenced areas, or unfenced ones separated by distance or by natural barriers [33].

We used information and samples from 1449 Iberian male red deer culled in 41 populations in the centre-west and centre-south of Spain between 2005 and 2019. All deer came from legal hunt during the hunting season (from October to February) under the modality of big game driven hunt, the traditional Spanish ‘montería’ [34, 35]. The regional governments of Extremadura and Andalucia granted permission for data and sample collection from hunted animals. No deer were culled specifically in order to carry out this study.

HC and LC population habitat included mountain ranges covered by Mediterranean shrub (*Cistus* spp., *Erica* spp., *Arbutus unedo*, *Phyllirea* spp., *Genista hirsuta*, *Lavandula* spp.) and tree species (*Quercus* spp., *Olea europaea*), along with lower, flatter land, covered by open oak woodland known as ‘dehesa’ [33, 36]. The information used in this study was average antler length, mandible length; age (in years); population; month when samples were collected and year.

Mandible length was measured in the laboratory from the mesial border of the first incisor socket to the vertical part of the ramus, after having removed the flesh at these two points. We used mandible length as a measurement of skeletal size body condition independent, to control for animal size [37, 38].

The average of antler length was measured on the animal carcass from the centre of the lowest outside edge of the burr over the outer side to the most distant point of the main beam, in both antlers. The point of the burr was where the centre line along the outer side of the beam intersected the burr. Measurements of broken antler beams were discarded.

The age in years was estimated by counting the milky-coloured cement layers on the root pad (assuming 1 layer per year of age) of the sectioned mesial section of M_1_, aided by a reflected light microscope at magnification x20 to x25 [39, 40]. When M_1_ was missing, or the cement layers were poorly defined, M_2_ was used and the age in years was estimated as the number of cement layers plus one. This age estimation technique provides an acceptable approximation to the actual age [37].

For the dark ventral patch, we measured its longitudinal size from the penis to the anterior limit of the stained surface. Previous studies have shown that the mechanism of pigmentation is based on the excretion of high amounts of norepinephrine excretion metabolite deposited on the fur by a urinating behaviour during the rut [28]. The hair tinted in one year does not maintain at least with similar aspect to next seasons, so the maximum length with dark hair reflects the area that has been pigmented during the current rutting season, and it was data that we used in the analyses.

### Statistical analyses

**Fig 1:**
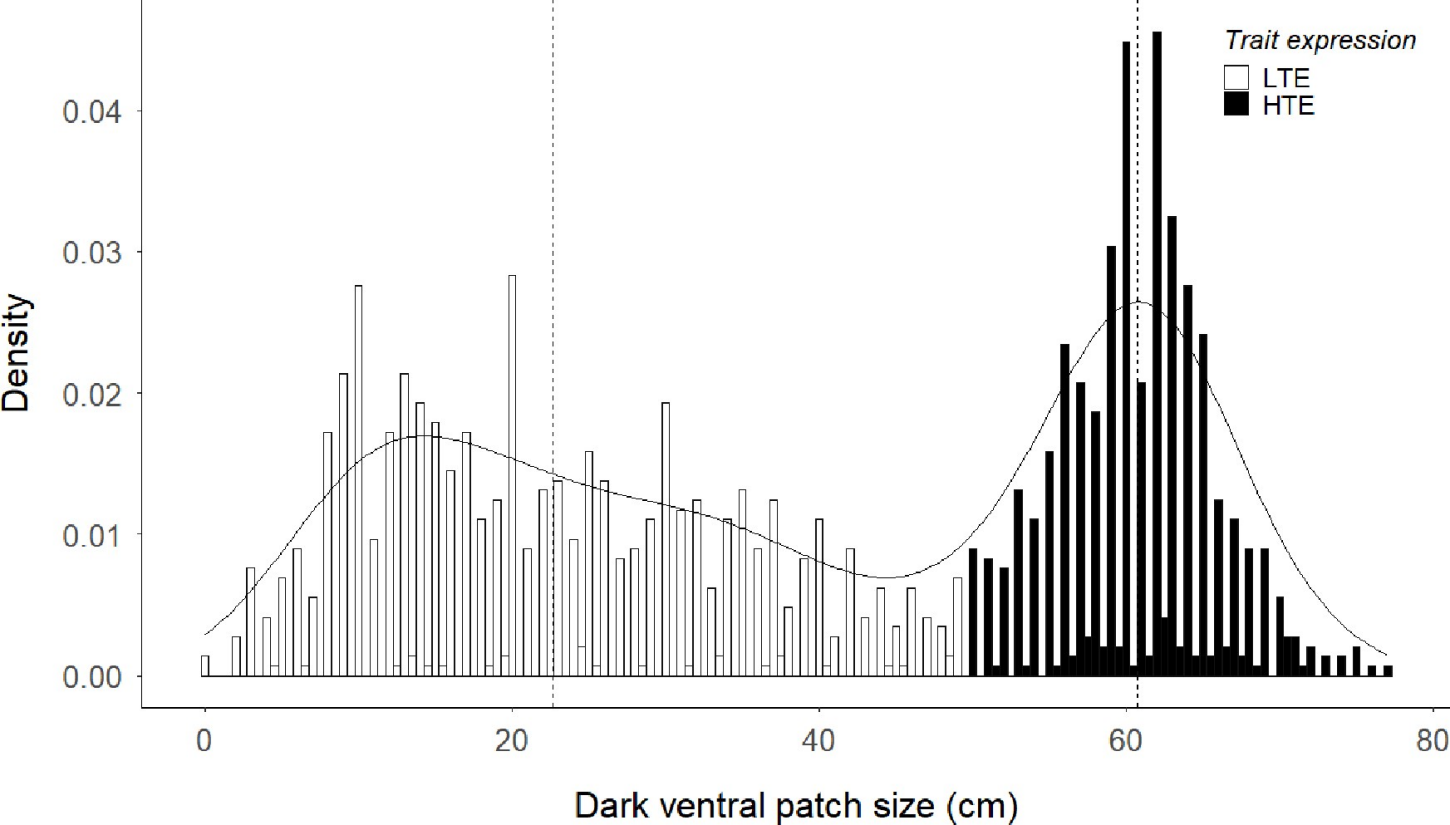
Density plot showing the bimodality distribution of the dark ventral patch size in male Iberian red deer. In black, high trait expression males (HTE), with patches larger than 50 cm. In white, low trait expression males (LTE), with patches between 0 cm to 50 cm.

Regarding the frequency histogram of the dark ventral patch size (see Fig 1), we checked for unimodality or multimodality, estimating the distribution underlying this trait by Hartigans’ dip test using the package diptest [41]. In previous assessments, the frequency histogram of the size of the dark ventral patch showed a data gap in values between 40 and 50 cm [42, 43]. Thus, we maintained here the cut point used in previous analyses that followed the distribution gap appearing in previous data sets, and defined two groups: low trait expression males, those that had between 0 and 50 cm of darkness area in their bellies, and those that had more than 50 cm, even the whole ventral area dark, high trait expression males.

To explore which variables were related to the size of the dark ventral patch we fitted a linear mixed model (LMM) fitted by restricted maximum likelihood (REML). This model included the following independent terms: both age and its quadratic term, antler length (cm) and mandible length (cm) as well as mate competition as factor with two levels (LC and HC). To account for variation among both groups of males, we added a new fixed effect predictor, dark ventral patch expression (a two-level factor as LTE and HTE but see Results). We also considered the two and three-way interactions, but we did not take into account the non-significant ones from the full model.

Because of the bimodality of the trait, we were interested in studying the variables that may have an effect in its expression, since they may reveal the information encoded in the potential sexual signal. For this purpose, we performed a generalized linear mixed model (GLMM) with the dark ventral patch expression as dependent discrete variable with two levels (LTE and THE) fitted to a binomial distribution. Independent terms were age, its quadratic term, mandible length, antler length and population level of mate competition.

In both models, to avoid risks of over-parameterization, we removed non-significant interactions sequentially (*p*-value > 0.05) following a backwards-stepwise selection procedure. We added “collection month nested into year”, “year” and “population” as random terms to control repeated sampling in the same population in different years and season that samples were collected. Models’ results were presented for all main effects and significant interactions. In the figures we present mean predicted values from the LMM and the probability values from the GLMM. Both models were tested in R v.2.14.0 (R Foundation for Statistical Computing, Vienna, Austria) using the package lme4 [44].

We checked the normal distribution of the model residuals, when explaining variation in the dark ventral patch size, using a Shapiro-Wilks test and we assessed the assumptions of homogeneity of variance plotting residual vs. fitted values. We also examined the presence of outliers and potential influential data points using Cook’s distance graphs. To avoid the multicollinearity between variables included in the analyses, we calculated the variance inflation factors (VIFs) [45] of the built model, using the R package *usdm* [46]. We did not find any evidence of collinearity (VIF < 2.226, see S1 and S2 Appendices). To facilitate model convergence, all quantitative variables were z-transformed, being the mean of zero and a standard deviation of one (using the scale function). The means are given ± SE and the level of statistical significance was p < 0.05. Predictions were visualized with ‘ggeffects’ [47] and ‘ggplot2’ (v3.1.1) was used for graphics [48]. Dataset used in this study is available at https://doi.org/10.6084/m9.figshare.12645821

## Results

The size of the dark ventral patch had less than 0.001% chance of being unimodal or a continuous trait (Hartigans’ dip test; D = 0.050, *p*-value < 0.001). It appeared as a bimodality character in a consistent way in different populations, among years and sampling dates (Fig 1).

Results from the LMM are shown in Table 1. We found a positive relationship between the dark ventral patch size and antler length, mandible length and age controlling for trait expression. We did not find any significant relationship between age quadratic term or male-male competition situation on the response variable.

**Table 1:** Factors influencing variability in the dark ventral patch size.

Term	Estimate	SE	Variance	SD	df	t-value	*p-value*
Fixed factors							
Intercept	43.762	0.882			15.550	49.575	**<0.001**
Trait expression (HTE)	-15.980	0.264			1440.370	-60.465	**<0.001**
Age	1.513	0.325			1402.190	4.650	**0.001**
Mate competition (HC)	0.096	0.411			37.290	0.235	0.817
Antler length	4.035	0.383			770.530	10.529	**<0.001**
Mandible length	0.809	0.312			1158.630	2.598	**0.010**
Trait expression × Age	2.503	0.313			1289.740	7.987	**<0.001**
Trait expression × Antler length	1.611	0.310			1433.130	5.188	**<0.001**
Random factors							
Collection month nested year			4.756	2.181			**<0.001**
Year			5.500	2.345			**0.021**
Population			2.602	1.613			**<0.001**
Residual			57.511	7.584			-
$R_{m}^{2}$ = 84.24%							
$R_{c}^{2}$ = 87.12%							

Results from a linear mixed model (fitted by REML). Only main effects and significant (P < 0.05) interaction terms are shown. As covariables age, antler length (cm) and mandible length (cm). Mate competition refers to a factor with two levels: low competition (LC) and high competition (HC). All covariables were standardized in the model. In order to control de bimodality of the dependent variable we include trait expression as a factor with two levels: low trait expression (LTE) and high trait expression (HTE).

**Fig 2:**
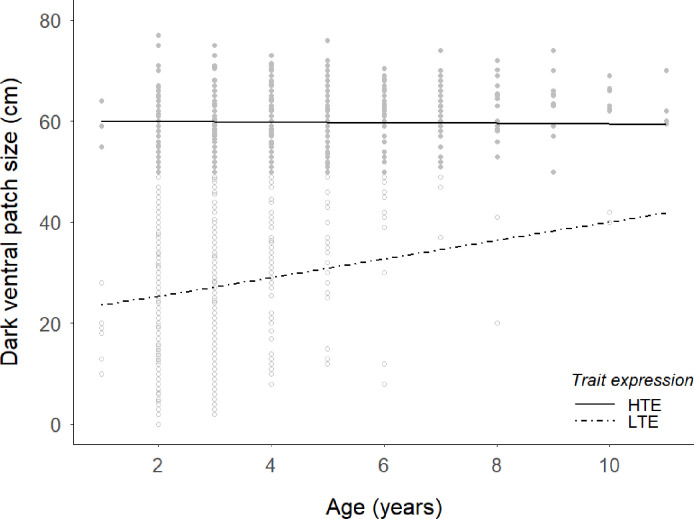
Predictions of the dark ventral patch size (cm) against age (years) from model of Table 1 for the two trait-expression groups of red deer males (HTE: solid line; LTE, dashed line). Points are raw, untransformed data for the dark ventral patch size (HTE: grey filled points; LTE, grey open points).

**Fig 3:**
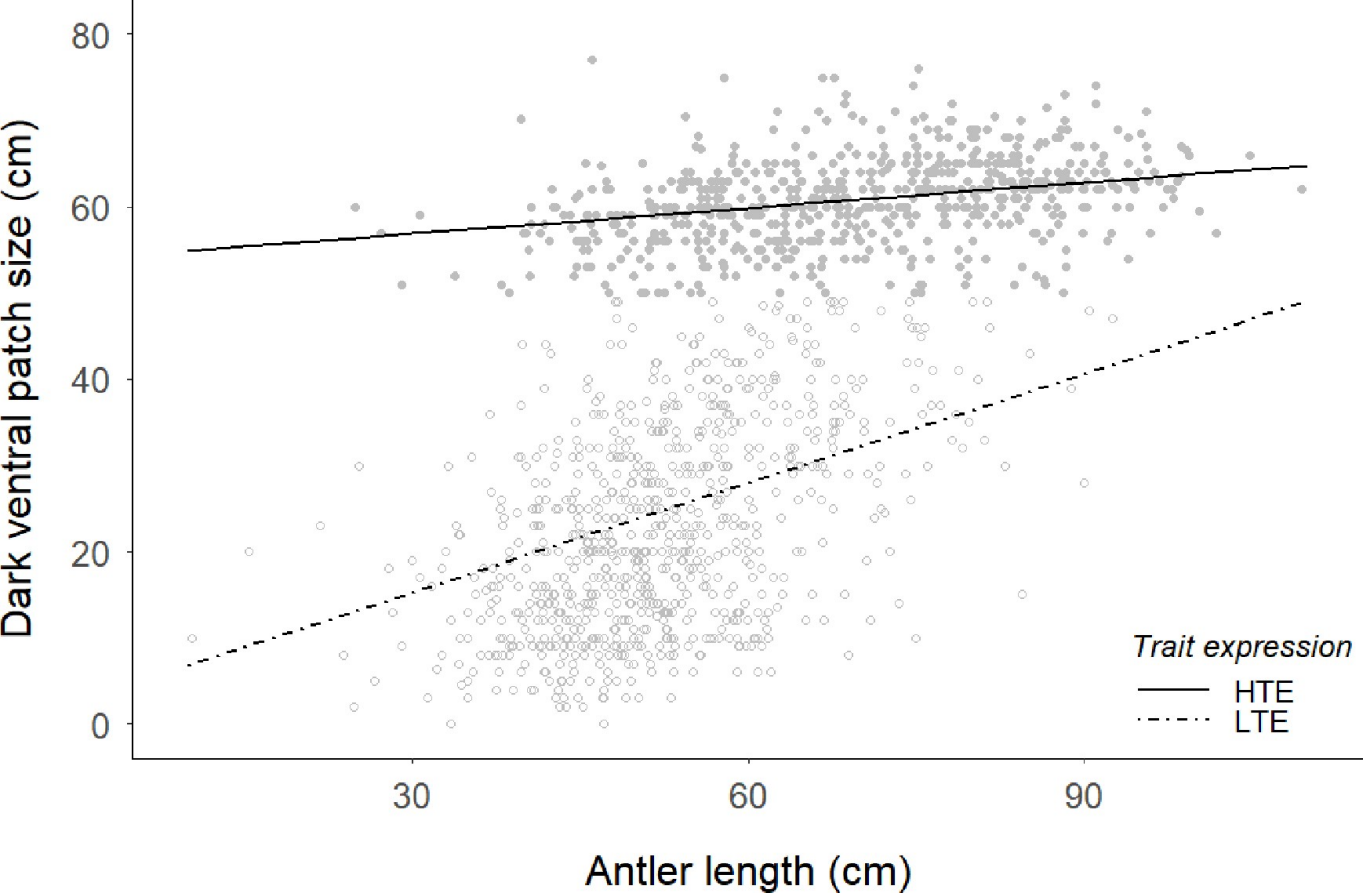
Predictions of the dark ventral patch size (cm) against antler length (cm) from the model on Table 1 for the two trait-expression groups of red deer males (HTE: solid line; LTE, dashed line). Points are raw, untransformed data for the dark ventral patch size (HTE: grey filled points; LTE, grey open points).

However, the interaction between trait expression and age revealed that high trait expression males presented patch sizes regardless of age, while low trait expression males showed a positive relationship between patch size and age (Fig 2). Likewise, the interaction term between trait expression and antler size indicated that only for LTE males, both variables were possitive related, while in HTE males trait size did not correlate with antler size (Fig 3).

**Fig 4:**
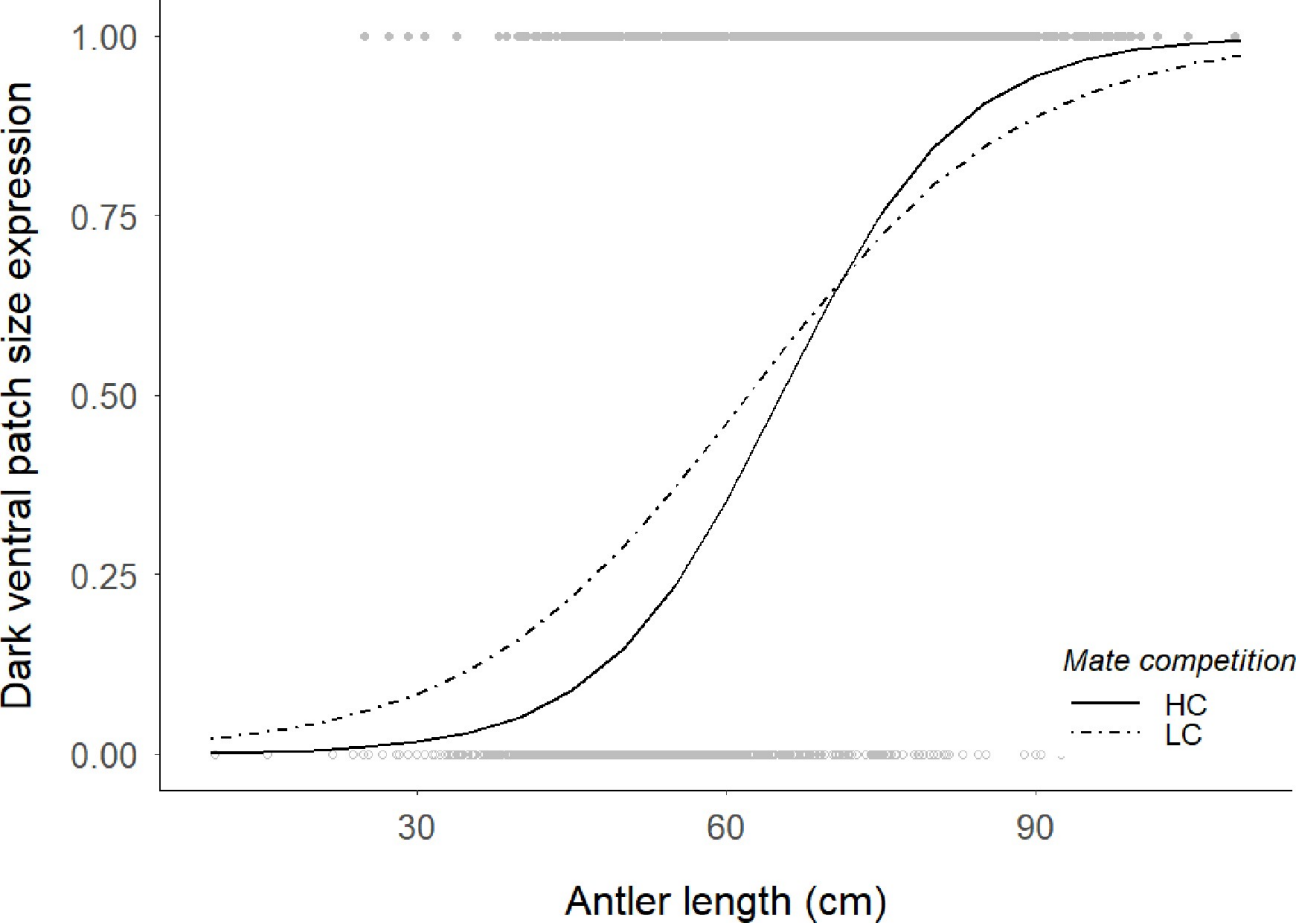
Predictions of the model of Table 2 on the dark ventral patch expression probability against antler length (cm) from populations with two levels of male-male sexual competition for mating opportunities (high competition, HC solid line, and low competition, LC dashed line). Points are raw, untransformed data for the dark ventral patch expression (1 = HTE: grey filled points; 0 = LTE, grey open points).

Derived from the GLMM (Table 2), both antler and mandible sizes had a significant effect in the probability to express a full sized dark ventral patch. But we also found differences between mate competition scenarios in the relationship between antler length and trait expression (interaction in Table 2). As male deer have relativey smaller antlers, individuals from low mate competition populations were more likely to express the trait than individuals from populations with high intrasexual competition. However, males with larger antlers had a greater probability of developing the traits when the mate competition was intense than when it was weak (Fig 4).

**Table 2:** Factors influencing the probability to express a full dark ventral patch.

Term	Estimate	SE	Variance	SD	Z-value	*p-*value
Fixed factors						
Intercept	-0.399	0.333			-1.201	0.229
Age	0.629	0.143			4.407	**<0.001**
Mate competition (HC)	0.230	0.177			0.301	0.193
Antler length	1.462	0.157			9.275	**<0.001**
Mandible length	0.417	0.117			3.563	**<0.001**
Antler length × Age	-0.252	0.122			-2.062	**0.039**
Mandible length × Age	-0.172	0.101			-1.711	0.087
Antler length × Mate competition						**0.022**
Random factors						
Collection month nested year			0.624	0.789		
Year			0.724	0.855		
Population			0.624	0.789		
$R_{m}^{2}$ = 42.26%						
$R_{c}^{2}$ = 63.64%						

Results from a generalized linear mixed model (fitted by ML) with a binomial distribution, and with LTE as reference level of the dependent variable, dark ventral patch expression. Only main effects and significant (P < 0.05) interaction terms are shown. As covariables age, age^2^, antler length (cm) and mandible length (cm). Mate competition refers to a factor with two levels: low competition (LC) and high competition (HC). All covariables were standardized in the model.

Moreover, we found that the interaction between antler length and age was significant, and we also showed a marginal effect of the interaction between age and mandible length on the trait expression. In other words, the link between both antler and mandible length was not the same among ages: when individuals had 6 years and above, regardless of the antler and mandible size, the probability of expression of the character was maximum.

## Discussion

Our results show for the first time the bimodal character of the dark ventral patch in male Iberian red deer. Variation in size of this trait among males in the population appeared related to antler size and age. However, these relationships can be noticed only for males not showing a fully expressed trait. For males developing the full trait, likely because it reached its asymptotic size, trait size appeared unrelated to age or antler development. Such a discrete nature of the trait, points to a potential role in signalling male situation in the context of competition for mates.

Antler length and mandible length (as a proxy of body size) appeared positively related to dark ventral patch size, although the relationships were blurred when the trait was fully developed. The observed age-related pattern of the dark ventral patch was similar to that of other secondary sexual traits in this species, such as antler length [49]. Our results showed that younger males showed a wide range of variation in the size of the dark ventral patch but that for males 7 years old or older, most of them presented the dark ventral patch of its asymptotic size. According to the mating-strategy-effort hypothesis [50], the maximum effort during the mating season is reached at prime-age, which in Iberian red deer is at 7–9 years old [51].

Many quality signals normally vary unimodally among individuals, due to the variability in the ability to cope with the associated costs of signal production and maintenance [52]. However, both empirical data and theoretical models [53] show that relationships between quantitative variables and their responses (input-output characteristics) are commonly non-linear. Sigmoidal-like relationships are common and lead to discrete responses from a continuous underlying cause of variation (see Fig 4 for antler size and ventral patch trait expression). This input-output pattern is in agreement with a signalling role of the trait [54, 55]. It is well known that discrete signals reduce errors [56] and, especially in aggressive interactions, animals predominantly use discrete signals while the underlying properties being signalled tend to be continuous [57, 58]. For instance, in lacertid species, it was documented a binomial expression of the differences in fight capacity between morphos, that were related to reproductive success (*Uta stanburiana* [59]; *Podarcis muralis* [60]).

Antler size is a highly costly secondary sexual character [61] used in fights during the intrasexual competition [62]. Antler size is related to male ability to winning contest [49, 63] and mating success [64]. Body size is also a good predictor of male fighting ability [65]. Both antler and body size are continuous traits, and the dark ventral patch may act as an amplifier for them [49] reducing the error in communication. Also, such a discrete nature of the ventral patch size suggests that trait expression not only provides information on male quality [27], but it may also reveal a behavioural strategy, like it has been shown for discrete signals in other species [66–68], of rival challenging, or willingness to be involved in competition for mates.

Contrary to other morphological traits, the dark ventral patch can be modulated in the short term by urine spraying [28], which suggests a fine tune in communication during the mating season compared to body or antler size. Previous research has shown that, due to the pigmentation mechanism mediated by a urine excretion metabolite of norepinephrine [28], the dark ventral patch size may contribute to rival assessment during male-male competition as well as reveal the reproductive effort of a male during the current rutting season. In addition, previous studies on the lipid compounds impregnating the hairs of this area show that this character may act as a chemical sexual signal [29] modulated by the competitive context [30].

Behavioural observations indicate that high trait expression males were more involved in rutting behaviours than low trait expression males [31]. Hence, large-patch males may be signalling their willingness and capacity for winning contests during intrasexual competition. On the other hand, involvement in behavioural interactions may entail maintenance costs for males expressing fully developed traits. Previous studies have shown differences in cortisol and testosterone levels related to trait size between the two male groups regarding trait expression (LC and HC) [69]. Additionally, there is also evidence that males with large patches were more susceptible to parasite infection than males with small patches [42].

Nevertheless, why some young males may show high expression trait during the rut is another issue. We expected that plasticity of the trait could allow individuals to invest in signalling and the associated reproductive effort according to their chances under the prevailing social conditions, i.e. the intrasexual competition level of the population. Previous studies showed that the chemical scent constituents in this trait are mediated by the social environment [30]. In this work we did not find evidence of the role of male-male competition *per se* on the dark ventral patch expression. However, the relationship between antler length and the probability of full trait expression was affected by the population level of male competition. Thus, males with relatively small antlers were more prone to develop full-size patches when in LC environments, while males with large antlers did so in HC environments. In HC environments males with small antlers have little chance of success [49, 63–65], so they should reduce signalling especially if there are social costs associated to the maintenance of the signal derived from interaction with more competitive males [52]. Under LC conditions, on the contrary, mature males with large antlers are scarce, so the chances of mating for smaller males are higher as shown by genetic data in these populations [32, 70] and also the possible costs of maintaining the trait may be lower due to the lower presence of rivals.

Our results as a whole indicate that the dark ventral patch expression is a discrete communicative signal [58] that reveals whether or not an individual is ready to invests in overt male-male competition during the breeding season, under a particular level of intrasexual competition in its social environment. The signal encodes information on antler and body size, which are features related to competitive ability [64, 65], as well as a physiological status (level of catecholamines and urinary excretion of their derivate DOPEG [28]). However, likely due to the non-linear relationships between condition features and the response [53], the dark ventral size becomes a discrete signal amplifying the communication of discrete, alternative strategies of male willingness to take part in mating competition in a given breeding season [55, 71].

Future work should explore the relationship between the expression of the dark ventral patch along life and male’s overall fitness. There are many examples of species in which certain zones susceptible to more rapid change, such as soft tissues instead of plumage in the case of birds, respond to a wide range of factors, not only endogenous such as body condition or health status, but also the social context [11, 15, 20, 72, 73]. Our results on the existence of two types of males regarding trait expression may help to understand the evolution of both trait trajectories under sexual selection and their covariance with the environment. Also, it is interesting to investigate the genetic mechanisms underlying the flexible expression of this character and the relationships with antler size and fitness, as well as to which extent the dark ventral patch is an honest and rapidly updated trait that adaptively responds to variations in both social and ecological environments [74].

## Supporting information

S1 Appendix Variance Inflation Factors (VIFs) for the explanatory variables included in the LMM explaining the differences in the dark ventral patch size considering trait expression age, antler length (cm), mandible length (cm) and mate competition. (DOCX)

S2 Appendix Variance Inflation Factors (VIFs) for the explanatory variables included in the GLMM explaining the differences in the dark ventral patch expression. (DOCX)
