# Supplementary material for: The dark ventral patch: A bimodal flexible trait related to male competition in red deer
Source: PLoS One. 2020 Nov 5;15(11):e0241374. doi: 10.1371/journal.pone.0241374 (PMC7644014; doi:10.1371/journal.pone.0241374)
Supplement: S1 Appendix — (DOCX) [file pone.0241374.s001.docx]

**S1 Appendix**. Variance inflation factors (VIFs) for the explanatory variables included in the LMM explaining the differences in the dark ventral patch size considering trait expression age, antler length (cm), mandible length (cm) and mate competition.

Trait expression = 1.405

Mate competition = 1.128

Age = 1.967

Antler length = 2.226

Mandible length = 1.568

Trait expression x Age = 1.744

Trait expression x Antler length = 1.443
