# Supplementary material for: The dark ventral patch: A bimodal flexible trait related to male competition in red deer
Source: PLoS One. 2020 Nov 5;15(11):e0241374. doi: 10.1371/journal.pone.0241374 (PMC7644014; doi:10.1371/journal.pone.0241374)
Supplement: S2 Appendix — (DOCX) [file pone.0241374.s002.docx]

**S2 Appendix**. Variance inflation factors (VIFs) for the explanatory variables included in the GLMM explaining the differences in the dark ventral patch expression.

Mate competition = 1.056

Age = 1.962

Antler length = 1.570

Mandible length = 1.248

Age x Antler length = 1.792

Age x Mandible length = 1.390

Mate competition x Antler length = 1.301
